# Detection of Alpha Fetoprotein Based on AIEgen Nanosphere Labeled Aptamer Combined with Sandwich Structure of Magnetic Gold Nanocomposites

**DOI:** 10.3390/bios13030351

**Published:** 2023-03-06

**Authors:** Lei Liu, Huixing Wang, Husseini Sulemana, Bing Xie, Li Gao

**Affiliations:** 1 Department of Kidney Transplantation, The Second Xiangya Hospital of Central South University, Changsha 410011, China; 2 School of Life Sciences, Jiangsu University, Zhenjiang 212013, China; 3 School of the Environment and Safety Engineering, Jiangsu University, Zhenjiang 212013, China; 4 The Fourth Affiliated Hospital of Jiangsu University, Zhenjiang 212001, China

**Keywords:** aptamer, AFP, detection, AIE

## Abstract

As a biomarker, alpha-fetoprotein (AFP) is valuable for detecting some tumors in men, non-pregnant women, and children. However, the detection sensitivity in some methods needs to be improved. Therefore, developing a simple, reliable, and sensitive detection method for AFP is important for non-malignant diseases. An aptamer binding was developed based on aggregation-induced emission luminogen (AIEgen) nanosphere labeled with Fe3O4@MPTMS@AuNPs. AFP was detected with a sandwich structure of AuNPs magnetic composite particles. An aggregation-induced emission (AIE) molecule and polystyrene (PS) nanosphere complex were assembled, enhancing the fluorescence and improving the sensitivity of detection. The limit of detection (LOD) was at a given level of 1.429 pg/mL, which can best be achieved in serum samples. Finally, the results obtained showed the complex to be promising in practical applications.

## 1. Introduction

Alpha-fetoprotein (AFP), the most commonly used protein biomarker, has attracted great attention recently. As a biomarker, AFP is valuable for detecting some tumors in men, non-pregnant women, and children. In addition, in high-risk but asymptomatic populations, AFP detection indicators are vital for identifying early curable tumors and reducing disease-related mortality in a cost-effective manner. The development of AFP detection technology with high sensitivity is necessary for the early detection of some cancers and the clinical detection of AFP [1,2,3,4]. To this end, a variety of methods for detecting AFP have been developed, such as enzyme-linked immunosorbent assay [5], radioimmunoassay [6], fluorescence immunoassay [7], electrochemiluminescence [8], Raman spectroscopy [9], electrochemical immunosensing [10], and so on. However, they possess the shortcomings of non-specific binding and are unsuitable for high-throughput analysis [11]. Antibodies are associated with poor reproducibility and instability, which limits their broad application. For example, some small molecules cannot react and antibodies are unstable in extreme environments. Furthermore, immunoassays can produce false-positive or false-negative results [12]. An aptamer is a single-stranded RNA or DNA molecule selected in vitro from the nucleic acid molecular library by systematic evolution of ligands by exponential enrichment (SELEX) to specifically combine targets with high affinity (nucleic acids, small molecules, proteins, etc.) [13]. Aptamers are flexible, repeatable, easy to fix and regenerate, and show no difference between batches, and have been widely used in the sensor field [14]. Since aptamers exhibit many beneficial properties, aptamer-based biosensor systems have been developed to analyze various classes of detectors. Therefore, developing rapid, highly sensitive, selective, and high-efficiency methods for detecting AFP using aptamers is essential for human disease diagnosis.

Microfabrication technology is widely used for manufacturing, such as for 3D printing, microfluidics, and in devices used for detection. However, it needs expensive production equipment [15]. As a material in the field of nanomaterials, magnetic nanomaterials have attracted more and more attention due to their unique magnetism, low toxicity, and good compatibility [16]. Research has shown that nanomaterials have potential applications in magnetic separation [17], medical imaging [18], drug targeting, and even cancer treatment [19]. Based on current research results, relevant studies have ascertained that, if two or more metal nanomaterials are synthesized into composites, they can not only ensure the essential properties of each metal, but also have the potential to enable expression of more properties, such as particular physical (such as adsorption) or chemical (active binding site) properties. Luminescent materials in fluorescent biosensors have been widely used in the environment, health, and energy fields due to their high energy conversion characteristics, good resolution and in situ processing [20,21,22]. Recently, researchers have found that coupling or adsorbing fluorescent molecules into nanoparticles can improve detection sensitivity [23]. Specifically, AIE-active molecules are almost nonemissive in dilute solution states. There is strong fluorescence emission in aggregated states, resulting in a turn-on fluorescent signal with better accuracy and higher accuracy sensitivity for the sensing platform [24]. Li et al. designed a silica nanoparticle based on AIE phosphor (AIEgen) [25]. According to morphological study, it was found that the silica precursor without fluorescence emitted strong fluorescence through fluorescence polymerization. On the other hand, the spatial structure inside polymer nanoparticles can limit the rotation of AIE molecules. AIEgen was assembled into nanoparticles that can also emit fluorescence. Studies have shown that AIEgen has been widely used for apoptosis.

DSAI is an AIE molecule combining a 9,10-distyrylanthracene (DSA) derivative with short alkyl chains as the florescent probe [23]. In this paper, the swelling method was used to enable AIEgen molecules (DSAI) and polystyrene (PS) nanoparticles to form AIEgen nanospheres. The surface of the fluorescent nanospheres was modified with chloroauric acid providing carboxyl functional groups and which covalently interacted with the amino-terminal modified aptamer. The spatial structure inside polymer nanoparticles can limit the rotation of AIE molecules. AIEgen was assembled into nanoparticles that can also emit fluorescence. At the same time, a new magnetic core shell was synthesized by 3-mercaptopropyl trimethoxysilane (MPTMS). AuNPs with Fe_3_O_4_ formed a core-shell composite structure, which provided a functional platform for magnetic nanomaterials and increased the binding with aptamers. Moreover, the magnetic Fe_3_O_4_ was separated from the buffer system by the action of a magnet, which made the experimental operation straightforward. 

## 2. Materials and Methods

### 2.1. Chemical Reagents and Experimental Materials

Aptamer 1, 5′-GTG ACG CTC CTA ACG CTG ACT CAG GTG CAG TTC TCG ACT CGG TCT TGA TGT GGG TCC TGT CCG TCC GAA CCA ATC-SH-3′.

Aptamer 2, 5′-NH_2_-GTG ACG CTC CTA ACG CTG ACT CAG GTG CAG TTC TCG ACT CGG TCT TGA TGT GGG TCC TGT CCG TCC GAA CCA ATC-3′.

3-mercaptopropyltrimethoxysilane (MPTMS) and sodium dodecyl sulfonate (SDS) were ordered from Sigma, Tokyo, Japan. Fe_3_O_4_ nanomaterials and polystyrene (PS) nanoparticles were ordered from the Suzhou derivative Biotechnology Co., Ltd., Suzhou, China, whereas dichloromethane was ordered from the Aladdin Reagent Co., Ltd., Shanghai, China.

### 2.2. Experimental Instruments

All fluorescence spectra and required absorbance were recorded under 428 nm excitation using a Bio-Tek synergy H_4_ multifunctional microplate reader made in the United States. A high-resolution transmission electron microscope (JEM-2100 (HR)) was used to produce TEM images at 200 KV. A vacuum freeze-dryer (FD-1A-50) was used to freeze-dry the samples at a given temperature of 53 °C with a vacuum at 20 Pa. Fourier transform infrared spectroscopy (FT-IR) was obtained from Perkin Elmer Inc using KBr to treat the sample. A PHS-25 digital viscometer was used to adjust the pH of the solution. The data obtained in the experiment were processed using Origin 8.0.

### 2.3. Preparation of Fe_3_O_4_@MPTMS@AuNPs

The AuNPs in this experiment were prepared using the classical Frens method [26]; the particle size was about 12 nm. A sol-gel method was used to synthesize Fe_3_O_4_@MPTMS@AuNPs according to Mohebbi et al. [27]. The synthesized gold nanoparticles were stored at a standby temperature of 4 °C. MPTMS, ultrapure water, pure ethanol, and hydrochloric acid were thoroughly mixed with a magnetic stirrer at a molar concentration ratio of 4:20:50:0.1 for 3 h. The solution was then placed in the chamber for 24 h and ultrasonically mixed with Fe_3_O_4_ in a volume ratio of 1:1, 2:1 and 4:1 for 30 min, washed many times, and lyophilized to obtain the solid Fe_3_O_4_@MPTMS. The sulfhydryl (-SH) was the surface of Fe_3_O_4_@MPTMS. This can further bind to Au NPs. After mixing the prepared gold nanoparticles with ethanol for 5 min, 0.1 g Fe_3_O_4_@MPTMS was added and stirred for 30 min. After washing and drying 3 times, Fe_3_O_4_@MPTMS@AuNPs were prepared into a solution of 2 mg/mL and homogenized by sonication for 5 min. 

### 2.4. Preparation of AIEgen

An amount of 100 mg polystyrene nanospheres was dissolved in 10 mL of ultrapure water (containing 0.25% SDS) to obtain a uniformly dispersed solution with ultrasound. A quantity of 0.5 mL of DSAI (mass fraction 10%) was dissolved in CH_2_Cl_2_ solution. After sonication for 1 min, it was stirred at 40 °C, and CH_2_Cl_2_ evaporated by rotation. The obtained AIEgen nanospheres were washed with ultrapure water 3 times to remove SDS, centrifuged at 8000 rpm for 10 min and washed with ethanol 3 times until fluorescence was not observed in the supernatant. Finally, the solid was dried using a freeze-dryer. A certain amount of solid was dissolved in ultrapure water to prepare a solution with a final concentration of 2 mg/mL and ultrasonic homogenization was applied [28].

### 2.5. Preparation of Carboxyl Functionalized AIEgen Nanospheres

Amounts of 0.6 g NaOH and 0.5 g chloroacetic acid were added to 5 mL of AIEgene nanosphere solution with a size of 50 nm, respectively. The solution was sonicated in a water bath for 2 h, then neutralized by NaOH and further purified by centrifugation at 8000 rpm for 10 min and washed 3 times. The solution was dried to obtain a carboxyl-functionalized AIEgene nanosphere.

### 2.6. Preparation of AIEgen Nanosphere Labeled Aptamer (AIEgen Aptamer)

AIEgene nanospheres were weighed and prepared as a solution with a 2 mg/mL final concentration. The solution was reacted with 30 µL (50 nM) aptamer 2 with amino (-NH_2_) functional group at 4 °C for 12 h and was subsequently centrifuged at 8000 rpm for 10 min to remove the excess aptamer 2 and dispersed in the buffer.

### 2.7. Sensor Fabrication Processing

**Figure 1:**
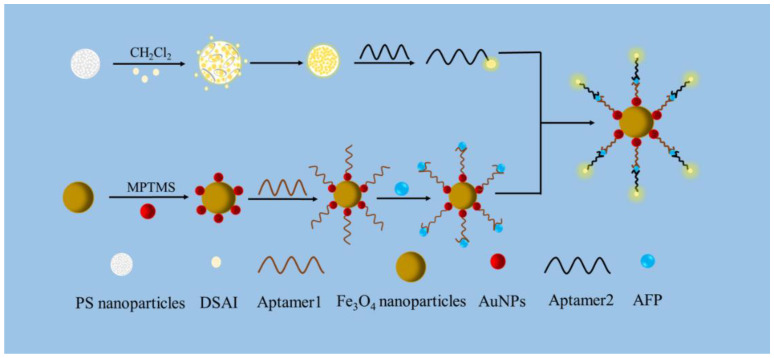
Schematic of alpha-fetoprotein detection by the sandwich structure of aptamer combined with ferromagnetic composite particles labeled with AIEgen nanospheres.

An aptamer that was based on AIEgen nanosphere labelling Fe_3_O_4_@MPTMS @AuNPs was developed in this study. AFP was detected using a sandwich-structure aptamer fluorescence sensor constructed with a combination of AuNPs and magnetic composites (Figure 1). Aptamer 1, with a final concentration of 15 nM, was mixed with 10 μg/mL Fe_3_O_4_@MPTMS@AuNPs. The mixture was incubated for 12 h in PBS (10 mM, pH 7.4) buffer with a total concentration of 500 μL and adsorbed with a magnet. Different concentrations of AFP were subsequently added and incubated at room temperature for 30 min. Consequently, AFP was explicitly bound to aptamer 1 and remained on the surface of Fe_3_O_4_@MPTMS @AuNPs, which prompted the addition of 8 μg/mL AIEgen aptamer 2, incubated at room temperature for 30 min. 

## 3. Results and Discussion

### 3.1. Sensor Detection Principle

The chemical binding between sulfhydryl (-SH) at the end of aptamer 1 and Fe_3_O_4_@MPTMS@AuNPs occurred on the surface of AuNPs with aptamer 2. The unbound AIEgen aptamer 2 was removed by a magnet. At this juncture, aptamer 1, AFP, and AIEgen aptamer 2 constituted a sandwich structure. AIE molecules and PS nanospheres were assembled into a complex state. The spatial structure inside polymer nanoparticles can limit the rotation of AIE molecules. AIEgen was assembled into nanoparticles that can emit fluorescence. If AFP does not remain on the surface of Fe_3_O_4_@MPTMS @AuNPs, AIEgen aptamer 2 should be quenched by Fe_3_O_4_@MPTMS @AuNPs. AIEgen aptamer 2 was far from the surface of Fe_3_O_4_@MPTMS@AuNPs based on FRET between Fe_3_O_4_@MPTMS@AuNPs and AIEgen aptamer 2 after adding AFP. Then an AIEgen fluorescent ball emitted the fluorescence. After the same elution, the fluorescence intensity was measured at this time. The measured values of the reaction system using the multifunctional enzyme-labelling instrument were processed by Origin 8.0, and the value of F/F_0_-1 was obtained. The value of F/F_0_-1 is the ratio of fluorescence intensity, where F_0_ and F, respectively, represent the fluorescence intensity of the fluorescence sensing system at 550 nm before and after the addition of AFP. 

### 3.2. Characterization of Fe_3_O_4_@MPTMS @ AuNPs and AIEgen Nanospheres

**Figure 2:**
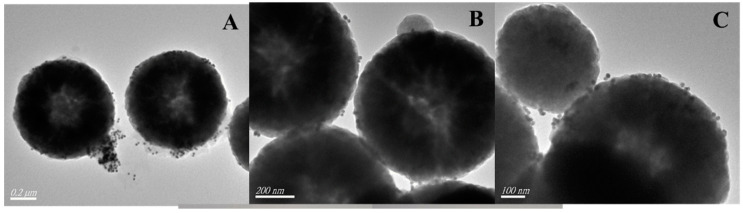
TEM images of Fe_3_O_4_@MPTMS@AuNPs synthesized by different volume ratios and Fe_3_O_4_@MPTMS@AuNPs. (**A**) It can be seen that there were still some AuNP particles in the composite system that were not completely bound to the surface of magnetic nanoparticles. (**B**) It can be seen that AuNPs were distributed on the surface of each magnetic nanosphere. The volume ratio of Fe_3_O_4_ to MPTMS was 1:2. (**C**) It was observed that AuNPs were unevenly distributed on the surface of Fe_3_O_4_ particles.

In the process of combining Fe_3_O_4_ with MPTMS, if the amount of MPTMS is too low, the effective binding rate of Fe_3_O_4_ with AuNPs will be reduced; if the amount of MPTMS is too high, it will make the MPTMS in the oil state difficult to clean. Therefore, we optimized the combination ratio of Fe_3_O_4_ and MPTMS based on literature reports. It can be intuitively observed from Figure 2 that the AuNPs nanoparticles were bound to the surface of Fe_3_O_4_ through the action of MPTMS. However, it can be seen from Figure 2A that some AuNPs in the composite system were not fully combined on the surface of magnetic nanoparticles. Figure 2C shows that AuNPs were unevenly distributed on the surface of Fe_3_O_4_ particles. In Figure 2B, AuNPs were distributed on the surface of each magnetic nanosphere. Therefore, a volume ratio of Fe_3_O_4_ to MPTMS of 1:2 was chosen to prepare subsequent composite nanomaterials. Figure 2C shows Fe_3_O_4_@MPTMS @AuNPs. A comparative image of the AuNPs solution and the solution after the action of the magnet shows that the magnet was able to completely adsorb the composite nanomaterials on the bottle wall and make the solution clear.

### 3.3. Fluorescence Spectra and Characterization of AIEgen Nanospheres

**Figure 3:**
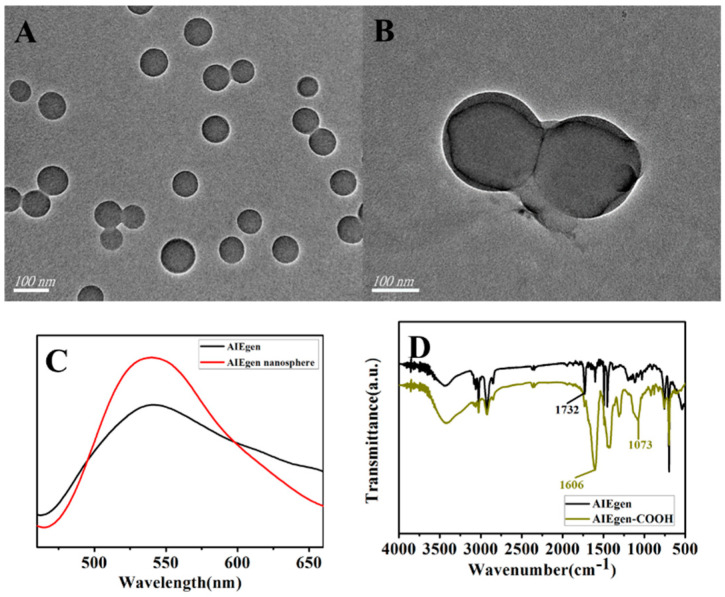
TEM and fluorescence spectra of AIEgen and AIEgen nanospheres as well as FT−IR spectra of carboxylated functionalized AIEgen. (**A**) and (**B**) show TEM diagrams of PS nanospheres and AIEgen nanospheres. (**C**) shows a comparison of the fluorescence spectra of AIEgen (DSAI) and AIE/PS assembled polymer. It can be seen from the figure that the fluorescence intensity of AIEgen assembled into PS nanospheres was enhanced. (**D**) shows the FT−IR spectrum of carboxyl functionalization of AIEgen. The peak at 1606 cm^−1^ represents the formation of carboxyl functional groups; a strong, broad peak appears at 1073 cm^−1^.

Figure 3A,B show the TEM images of PS nanospheres and AIEgen nanospheres, respectively. Figure 3C compares the fluorescence spectra of AIEgen (DSAI) and AIE and PS assembled into polymers. The fluorescence value was enhanced when AIEgen was assembled into PS nanospheres. Figure 3D shows the FT-IR spectrum of carboxyl functionalization of AIEgen. The peak at 1606 cm^−1^ represented the formation of carboxyl functional groups, and there was a strong, wide peak at 1073 cm^−1^.

### 3.4. Optimization of Experimental Conditions

The experimental conditions were optimized in this study to achieve the best detection effect of AFP on the sensing platform, including the concentrations of Fe_3_O_4_@MPTMS@ AuNPs, aptamer 1, and AIEgen aptamer 2.

#### 3.4.1. Optimization of Fe_3_O_4_@MPTMS @ AuNPs Concentration

**Figure 4:**
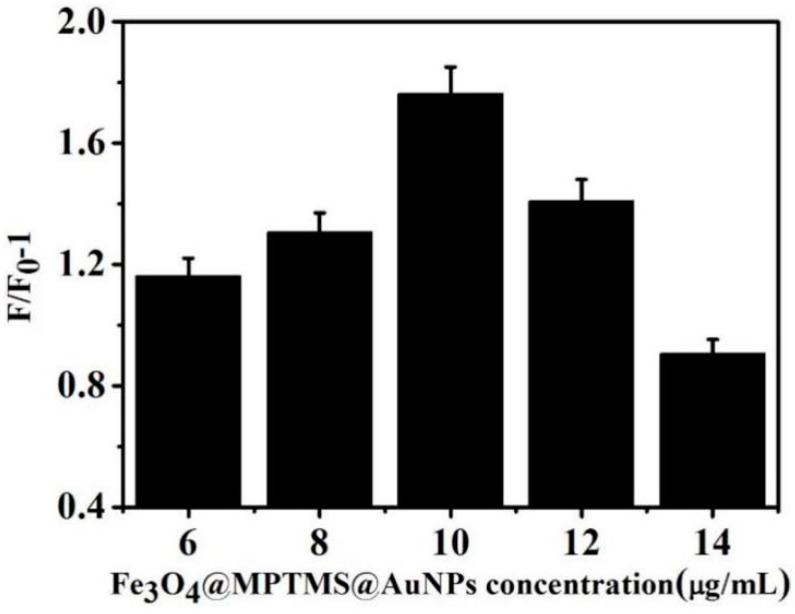
Effect of different concentrations Fe_3_O_4_@MPTMS@AuNPs on fluorescence intensity.

The influence of AuNPs on the reaction system was optimized. Different concentrations of Fe_3_O_4_@MPTMS@AuNPs (6, 8, 10, 12 and 14 μg/mL) were added to the buffer system containing 15 nM aptamer 1. The fluorescence intensity of the system was measured before and after adding AFP, and the change in fluorescence intensity at 550 nm was compared. The results are shown in Figure 4. The results showed that F/F_0_-1 gradually increased with the low concentration of Fe_3_O_4_@MPTMS@AuNPs nanocomposites. When its concentration was 10 μg/mL, F/F_0_-1 reached a maximum value. When the concentration of nanomaterials continued to increase, F/F_0_-1 gradually decreased. Therefore, the study used 10 μg/mL Fe_3_O_4_@MPTMS@AuNPs complex as the optimal concentration.

#### 3.4.2. Optimization of Aptamer 1 Concentration

**Figure 5:**
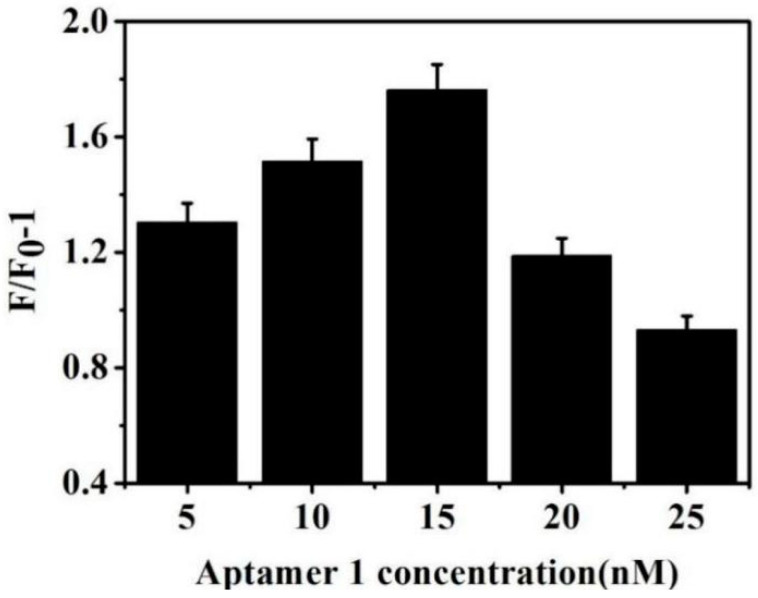
Effect of different concentrations of aptamer 1 on fluorescence intensity.

The concentration of aptamer 1 was optimized. Amounts of 5, 10, 15, 20, and 25 nM aptamer 1 were added to the reaction system. Very low concentrations of aptamer 1 in the system affected Fe_3_O_4_@MPTMS@AuNPs binding and immobilization for AFP. However, it was not necessary at very high concentrations of aptamer 1. Figure 5 shows the highest F/F_0_-1 caused by aptamer 1 with a concentration of 15 nM under the same conditions. Consequently, aptamer 1, with a concentration of 15 nM, was chosen as the optimal concentration in this study.

#### 3.4.3. Optimization of AIEgen Aptamer 2 Concentration

**Figure 6:**
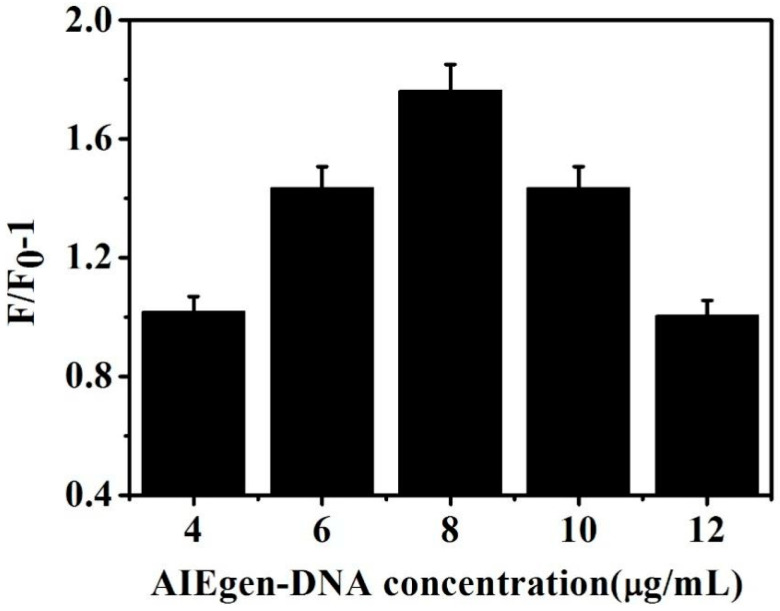
Effects of different concentrations of aptamer AIEgen-aptamer 2 on the fluorescence intensity.

The influence of AIEgen aptamer 2 on the fluorescence sensor system was further explored and optimized. The sandwich structure system added different concentrations (4, 6, 8, 10, and 12 μg/mL) of AIEgen aptamer 2. Figure 6 shows that, with gradual increase in AIEgen aptamer 2 concentration, F/F_0_-1 gradually increased. When the AIEgen aptamer 2 concentration increased to 8 μg/mL, F/F_0_-1 reached a maximum value. Moreover, the subsequent fluorescence change value gradually decreased with increase in AIEgen aptamer 2 concentration. However, if the concentration of aptamer 2 was more than 8 μg/mL, it was unnecessary. A higher concentration of aptamer 2 was able to increase the intensity of the fluorescence background. Nevertheless, the effect of detection was not improved. F/F_0_-1 did not increase with a higher concentration of aptamer 2, prompting the selection of 8 μg/mL as the optimal concentration.

### 3.5. Sensitivity Test

**Figure 7:**
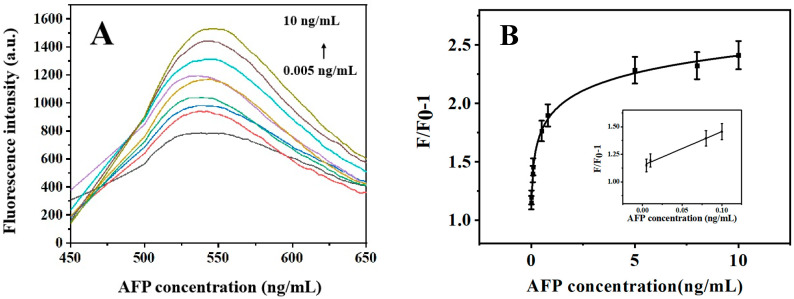
Fluorescence intensity (**A**) and F/F_0_-1 (**B**) changes induced by different concentrations of AFP.

Different concentrations of AFP (0.005, 0.01, 0.08, 0.1, 0.5, 0.8, 5, 8, and 10 ng/mL) were added to the solution containing 10 μg/mL Fe_3_O_4_@MPTMS in the sensing system of AuNPs; amounts of 15 nM aptamer 1 and 8 μg/mL AIEgen aptamer 2 were mixed evenly and eluted after incubation for 30 min at room temperature. The changes in fluorescence were recorded and calculated under different concentrations of AFP. The results are shown in Figure 7. Higher concentration of AFP can bind to more aptamer with DSAI. Therefore, the fluorescence intensity value gradually increased with increase in AFP concentration. The AFP concentration was in the range of 0.005–0.1 ng/mL as shown in Figure 7B; F/F_0_-1 exhibited an apparent linear relationship with AFP concentration; the linear regression equation was y = 3.082 × C[AFP] + 1.148, R^2^ = 0.99. As the AFP concentration increased, the fluorescence intensity increased relatively slowly. We calculated LOD based on signal-to-noise (LOD = 3S/N), where S is the standard deviation of the eleven blank measurements (without AFP), and N is the slope of the fluorescence intensity of DSAI relative to the AFP concentration. Based on 3S/N, the detection limit was 1.429 pg/mL. As shown in Table 1, this sensor had a lower LOD and an excellent linear range. It was better than some reported sensors. The limit of quantification (LOQ, ten times of standard deviation of the blank signal/slope) was 4.873 pg/mL.

### 3.6. Selective Detection

**Figure 8:**
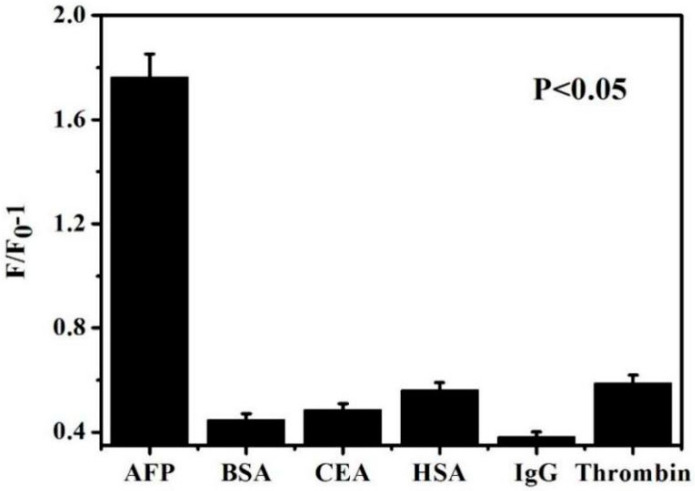
Effects of AFP and interferences BSA, CEA, HSA, IgG, and thrombin on the sensor under optimal conditions.

Several substances similar to AFP, such as BSA, CEA, HSA, IgG, and thrombin, were introduced into the sensing system for interference experiments. Under the optimal conditions, AFP and other analogues with the same concentration (0.5 ng/mL) were added. The corresponding fluorescence intensity value and F/F_0_-1 were recorded. The result is shown in Figure 8. The same substance concentration was added under the same conditions. The fluorescence intensity of the sensor could be distinguished from others. This showed that the AFP detection using the sensor had good specificity.

### 3.7. Serum Sample Analysis

To prove the clinical stability of the sensor, we replaced the reaction system with a sensing platform based on serum. The serum was bought from Tianhang Biotechnology Co., Ltd. (Huzhou, China). The obtained information from AFP added in serum was detected using the biosensors. Three different concentrations of AFP (0.01 ng/mL, 0.08 ng/mL and 0.1 ng/mL) were selected in the linear range for standard recovery testing in normal serum. Three repeated experiments were carried out in each group. As shown in Table 2, the recoveries of the three groups of samples were 96.69%, 103.18%, and 105.224%, respectively. After calculation, the relative standard deviation was 1.856%–4.484%, which met the practical application requirements. A series of measurements from the batch resulted in a relative standard deviation (RSD) of 4.484%, demonstrating that the sensor results were repeatable and reproducible.

## 4. Conclusions

In this study, an aptamer based on AIEgen nanosphere labeled with Fe_3_O_4_@MPTMS@AuNPs was developed. The detection of alpha-fetoprotein by the sandwich structure of AuNPs magnetic composite particles was carried out. The unbound AIEgen aptamer 2 was removed by a magnet. AuNPs with Fe_3_O_4_ formed a core-shell composite structure, which provided a functional platform for magnetic nanomaterials and increased the binding with aptamers. At the same time, the magnetic Fe_3_O_4_ was obviously separated from the buffer system by the action of the magnet, which enabled a straightforward experimental operation. Therefore, this can specifically detect AFP. The AIE molecules and PS nanospheres were assembled into a complex. The spatial structure inside the polymer nanoparticles limited the rotation of the AIE molecules. AIEgen was assembled into nanoparticles and emitted fluorescence after aptamer 2 was bound to AFP, which caused aptamer 2 to be far from the surface of Fe_3_O_4_@MPTMS@AuNPs based on FRET because Fe_3_O_4_@MPTMS@AuNPs was able to quench the fluorescence of AIEgen. This enhanced the fluorescence intensity and improved the sensitivity of detection. Moreover, the detection limit using this method was 1.429 pg/mL, which was lower than LOD for most reported methods.

## Figures and Tables

**Table 1:** Comparison of the AFP detection methods.

Method	Analyst	Linear Range	LOD	Reference
Surface-Enhanced Raman Scattering (SERS)	AFP aptamer/IgG	50~100 ng/mL	50 pg/mL	[29]
Fluorescence Resonance Energy Transfer (FRET)	AFP aptamer/QDs-AuNPs	0.5~45 ng/mL	400 pg/mL	[30]
FRET	FAM-labeled AFP aptamer/PdNP	5~150 ng/mL	1.38 ng/mL	[31]
Cyclic Voltammetry (CV)	AFP aptamer/PBNPs	0.01~300 ng/mL	6.3 pg/mL	[32]
CV	AFP aptamer/TH/RGO/AuNPs	0.1~100.0 μg/mL	0.050 μg/mL	[33]
Electrochemical Impedance Spectroscopy (EIS)	Methyl orange doped polypyrrole	10~10^4^ pg/mL	3.3 pg/mL	[34]
EIS	Aptamer/graphene oxide	0.01~100 ng/mL	3 pg/mL	[8]
FRET	Aptamer/sandwich structure of AuNPs magnetic composite	0.005–0.1 ng/mL	1.429 pg/mL	This work

**Table 2:** Determination of AFP in the serum (*n* = 3).

Samples	Added	Obtained	Recovery	RSD
	(ng/mL)	(ng/mL)	(%)	(%)
1	0.01	0.00967	96.69	1.863
2	0.08	0.0825	103.18	4.484
3	0.1	0.105	105.224	1.856

## Data Availability

Not applicable.
